# Skin Lesion Segmentation Using Deep Learning with Auxiliary Task

**DOI:** 10.3390/jimaging7040067

**Published:** 2021-04-02

**Authors:** Lina Liu, Ying Y. Tsui, Mrinal Mandal

**Affiliations:** Department of Electrical and Computer Engineering, University of Alberta, Edmonton, AB T6G1H9, Canada; lina1@ualberta.ca (L.L.); ytsui@ualberta.ca (Y.Y.T.)

**Keywords:** skin lesion segmentation, convolutional neural networks, auxiliary task learning, edge prediction

## Abstract

Skin lesion segmentation is a primary step for skin lesion analysis, which can benefit the subsequent classification task. It is a challenging task since the boundaries of pigment regions may be fuzzy and the entire lesion may share a similar color. Prevalent deep learning methods for skin lesion segmentation make predictions by ensembling different convolutional neural networks (CNN), aggregating multi-scale information, or by multi-task learning framework. The main purpose of doing so is trying to make use of as much information as possible so as to make robust predictions. A multi-task learning framework has been proved to be beneficial for the skin lesion segmentation task, which is usually incorporated with the skin lesion classification task. However, multi-task learning requires extra labeling information which may not be available for the skin lesion images. In this paper, a novel CNN architecture using auxiliary information is proposed. Edge prediction, as an auxiliary task, is performed simultaneously with the segmentation task. A cross-connection layer module is proposed, where the intermediate feature maps of each task are fed into the subblocks of the other task which can implicitly guide the neural network to focus on the boundary region of the segmentation task. In addition, a multi-scale feature aggregation module is proposed, which makes use of features of different scales and enhances the performance of the proposed method. Experimental results show that the proposed method obtains a better performance compared with the state-of-the-art methods with a Jaccard Index (JA) of 79.46, Accuracy (ACC) of 94.32, SEN of 88.76 with only one integrated model, which can be learned in an end-to-end manner.

## 1. Introduction

The skin is the largest organ of the human body. When the skin cells become disordered and grow out of control, they can develop into skin cancers and may spread to other body parts. Skin cancer is the most prevalent cancer worldwide. Among all the types of skin cancers, melanoma is the most aggressive kind of skin cancer, whose incidence has risen rapidly over the last 30 years [1]. The best way to treat melanoma is its detection at an early stage. Specifically, the five-year relative survival rate for melanoma is 98% for the localized stage and drops to about 14% in the latest stage. Therefore it is critical to detect melanoma in a timely and accurate manner. To detect melanoma or the suspected skin lesions, dermoscopy imaging is used to detect the pigmented skin lesions. It is a non-invasive technique and is used as a primary step for the detection of suspected skin lesions. The dermoscopic images have a high resolution and enhanced visualization ability, which allow dermatologists to examine the skin lesions with naked eyes. However, the decision process is tedious, requires a great depth of expert knowledge, and is biased towards different dermatologists’s interpretation. Previous research has shown that melanoma detection based on convolutional neural networks (CNN) can obtain performance on par with that of dermatologists’ [2], which implies the potential for automatic skin lesion analysis. In addition, with the recent advances in image capturing and processing capabilities in smartphones, the acquisition of dermoscopic images using cellular phones has become very popular. Together with the automatic analysis methods, it can be a powerful tool that can provide a user-friendly intelligent interface and a possible telemedicine solution for melanoma screening outside the clinic [3,4]. Therefore, automatic analysis of skin lesions has become an important step in computer-aided diagnosis [5].

Numerous clinical metrics based on the appearance of local color and texture patterns for the detection of melanoma have been proposed using dermoscopy images, such as ABCDE rules [6,7], seven-point checklist [8] and classical pattern analysis [9]. The ABCDE rules provide an easy and general framework for dermatologists and patients to identify potential melanoma, which are defined as Asymmetry, Border irregularity, Color that is not uniform, Diameter greater than 6mm, and Evolving lesions (size, shape, or color). Typically, the borders of melanoma tend to be uneven and may have scalloped or nothed edges, that are vaguely defined. Therefore, skin lesion segmentation is usually performed first in order to get the boundary information or regions of interest (ROI), which has been proved beneficial for the subsequent classification or detection task [10,11].

**Figure 1:**
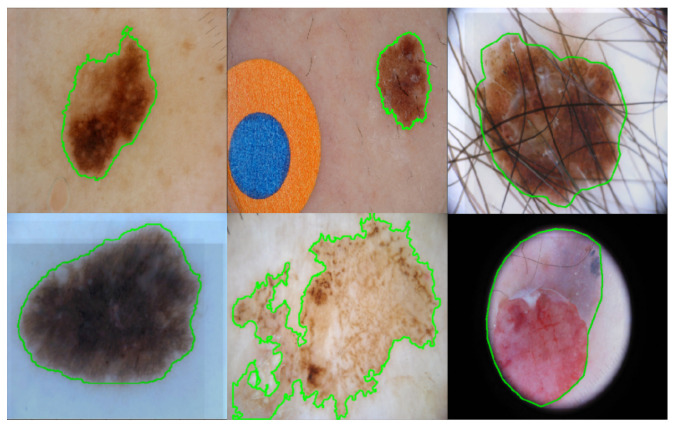
Some example images from the ISBI2017 dataset for skin lesion segmentation. The ground truth segmentation masks are marked using green contours. Fuzzy boundaries and distractions such as hairs and color-marks are also witnessed.

Automatic skin lesion segmentation is still a challenging task. For some skin lesions with light pigment, the color and visual patterns of the pigment regions and the surrounding skin regions are very similar, resulting in fuzzy and unclear boundaries, which makes the skin lesion segmentation task extremely difficult. In addition, the original dermoscopic images are of high-resolution, which is resource-intensive and time-consuming for the computers to process directly. Therefore, down-sampling is used first to reduce the image size. Finer textures and subtlety are lost during this procedure, which makes it even harder to differentiate the boundaries of these skin lesions. Moreover, the skin lesions also contain items such as hairs, veins, color-makers, rulers and glues, which affect the color and texture distribution of the skin lesions and impede successful learning. Figure 1 displays some example images from the ISBI2017 dataset [12] for skin lesion analysis, where the ground truth segmentation masks are marked using green contours. ISBI2017 dataset is one of the most challenging datasets for skin lesion segmentation tasks. The images are collected over different institutes and hospitals, and thus with different characteristics. As shown in Figure 1, the boundaries of some skin lesion images are very fuzzy and the pigment regions may share different visual patterns within the ROI. Hairs and color-makers are also observed among some images, which adds to the difficulty of skin lesion segmentation. The aforementioned problems make skin lesion segmentation a challenging task. To address these problems, literature works that deploy different CNN architectures with multi-scale information [11,13,14], or multi-task learning framework [15,16] have been proposed for skin lesion segmentation. The core idea of these methods can be regarded as trying to use as much information as possible to make robust predictions. However, these strategies either introduce extensive extra parameters for training or require extra labeling information, which may be inapplicable in practical situations.

In this paper, a novel CNN method that uses auxiliary information is proposed for skin lesion segmentation. The proposed method can be trained in an end-to-end manner without any pre-processing or post-processing steps. The contribution of this work is two-fold:

- Edge prediction is leveraged as an auxiliary task for the skin lesion segmentation task. The proposed method learns these two tasks simultaneously by two parallel branches (edge prediction and segmentation mask prediction). The edge prediction branch can guide the learned neural network to focus on the boundaries of the segmentation masks. Up to the authors’ knowledge, this is the first work that utilizes edge information to assist the skin lesion segmentation task. Note that the edge of a segmentation mask can be obtained automatically by applying some contour detection methods and hence no extra labeling effort is required for the proposed method.
- A cross-connection layer (CCL) module and a multi-scale feature aggregation (MSFA) module are proposed in this paper. The interaction of different tasks is realized by the CCL module. During the training process, the CCL module can implicitly guide the learning of the two tasks jointly, and hence boost each task’s performance in turn. Meanwhile, the MSFA module can make use of multi-scale information. Typically, a prediction head is placed at the intermediate feature maps of each resolution for both the edge prediction and segmentation prediction branch. The weights for the feature maps of each resolution can be learned automatically during training.

The organization of the paper is as follows. Section 2 presents a review of the related literature. Section 3 describes the proposed technique in detail. The experiment setup and performance evaluation of the proposed technique is presented in Section 4. Analysis of the results is presented in Section 5, followed by the conclusions in Section 6.

## 2. Related Works

Various skin lesion segmentation methods have been developed in literature, the conventional methods include the thresholding-based methods [17], region-merging based approaches [18], and active contour models [19,20,21]. Many traditional methods using morphological operations along with the clustering techniques [22,23] have been proposed. Jafari et al. [22] used K-means clustering to segment the skin lesion into the foreground and background region. Similarly, Ali et al. [23] proposed to use Fuzzy C-means (FCM) to perform skin lesion segmentation. Another popular class of approaches is the active contour models [19,20,21], where the contour can evolve iteratively toward the boundaries of pigment regions. After getting the candidate regions using thresholding methods, active contour models driven by local histogram fitting energy [20] or multi-direction gradient vector flow (GVF) snake [21] could be used to refine the course segmentation. However, traditional methods usually use complex pre-processing/post-processing techniques and may involve many intermediate steps, which are data-dependent. Therefore, the performance of traditional methods is highly dependent on these steps, and needs careful design when dealing with different datasets. They will fail when the boundaries of pigment regions are fuzzy and the skin conditions are complex.

The deep CNN models have achieved remarkable success in various computer vision tasks [24,25,26,27], and have also achieved the state-of-the-art performance for the skin lesion segmentation task. The basic CNN models generally use a sequence of convolution and pooling operations. As the neural networks go deeper, more semantic and abstract features (e.g., parts and shape) can be extracted using the learned kernels. Typically, for a classification neural network, the size of the output feature maps gradually decreases (by subsampling). The output is a probability vector with values in the range [0,1], whose dimension equals the number of categories. This path can be called an encoding path, where the images are encoded with semantic and abstract features as the neural network goes deeper. The overall structure for the segmentation network is very similar to the classification neural network, but usually with a decoding path, which aims at increasing the resolution of output (by upsampling) so that the size of the output segmentation mask equals the size of a given input image.

Based on the aforementioned introduction, the idea of treating the segmentation as a classification task has been proposed for skin lesion analysis by Jafari et al. [28]. The inputs are image patches of different scales centered at a certain pixel, and the ouput is the prediction label of this pixel. In this case, the local context information of the pixel is taken into consideration. However, this requires dense prediction as the proposed method was based on the pixel-level prediction and more recent works use CNN with a decoding path for the segmentation task. Ronneberger et al. proposed the well-known U-net [29], which is very popular due to its success on medical image segmentation tasks. Some methods based on the U-net [30,31,32,33] for melanoma segmentation and classification have been proposed. For instance, Liu et al. utilized dilated convolution at the end of each convolutional block of the original U-net to increase the receptive field of the proposed method. Abhishek et al. [33] incorporated and selected different color bands based on color transformations to further enhance the performance. Yuan et al. [34] proposed a method based on the fully convolution-deconvolution method. A loss function based on the Jaccard distance is used instead of the regular cross-entropy loss. Al-masni et al. [35] proposed a full resolution convolutional neural network, where the proposed method directly learned the full resolution features of each individual pixel of the input data by not using the sub-sampling operation. Bi et al. [36] trained independent CNN model for each known class to leverage the category information. A step-wise integration (PSI) model based on the hierarchical evolving model was used to refine the segmentation output. Sarker et al. [37] used dilated residual network with the pyramid pooling networks for skin lesion segmentation. The combination of negative log-likelihood and endpoint error loss is used to obtain sharp boundaries. Recently, Xie et al. [16] proposed a mutual bootstrapping CNN method that performs the skin lesion segmentation and classification simultaneously, and each task facilitates the other in a bootstrapping way. More specifically, a coarse segmentation network is trained, and the predicted coarse mask is used to guide the classification network. At the same time, class-specific localization maps generated via the classification activation mapping (CAM) are concatenated into a U-Net-like network for the prediction of the enhanced mask, which is more accurate than the coarse mask.

A recent technique named DEXTR (Deep Extreme Cut) [26] has shown that combining the extreme points (corner points on the contours) with the original RGB images as the input of CNN can improve the performance of instance segmentation of nature scenes [26]. However, [26] requires the input of extreme points, and the segmentation performance is dependent on the quality of these points. Subsequent works have shown that the auxiliary task, boundary/edge prediction, can help the instance segmentation task [38,39]. Based on this motivation, an automatic skin lesion segmentation method that uses edge prediction as an auxiliary task is proposed in this paper. Different from [38,39], a novel architecture that is based on CCL and MSFA module is proposed in this paper. Details about the proposed method are introduced in Section 3.

## 3. Methodology

**Figure 2:**
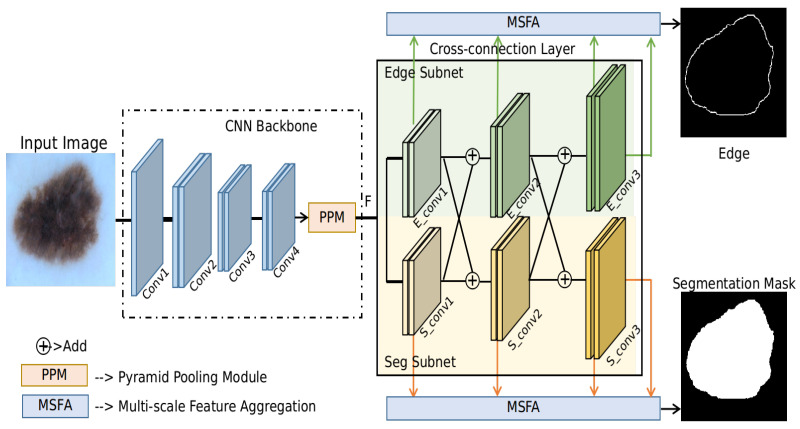
Schematic diagram for the proposed method. Edge prediction is used as an auxiliary task to help the segmentation task. Two parallel branches with the cross-connection layer (CCL) module are implemented so that the two tasks can interact with each other and boost each other’s performance in turn. A multi-scale feature aggregation (MSFA) module is used to aggregate information from feature maps of different scales.

In this section, details of the proposed method are presented. Given an input skin lesion image, the proposed method will simultaneously predict the segmentation mask and its corresponding edge (contour) during training. During the testing phase, only the segmentation mask is used for prediction. A schematic diagram of the proposed method is shown in Figure 2. It is observed that there are three main modules: CNN backbone, CCL and MSFA modules. Details of these modules are introduced in the following sections.

### 3.1. CNN Backbone

As shown in Figure 2, an input image first goes through a CNN backbone structure to generate the intermediate feature maps *F* for the subsequent edge prediction and segmentation mask prediction. In this paper, ResNet-101 [24] and Pyramid pooling module (PPM) [40] are used as the backbone structure for the proposed method, which can be regarded as a strong baseline model for the skin lesion segmentation task. The input images are resized to 448×448 before being fed into the CNN. To obtain a reasonable feature resolution for *F*, we modify the Conv4 layer in the ResNet-101 with a stride of 1, dilate rate of 2 following [26]. In this case, the resolution of the Conv4 layer will not decrease. Details about the architecture of the proposed method are shown in Table 1. Especially, [1×1,64] indicates the filter size is 1×1, and the number of filters (which equals to the number of output feature maps) is 64. [.]×3 means the operation inside the block is applied three times sequentially. Stride is set to be 1 and zero-padding is performed so that the resolution of the output feature maps remains the same. The PPM module [40] can utilize the context information of different resolutions and has been widely used as a plug-and-play tool. The same setting as [40] has been used, except that we had set the number of output channels to be 128. Specifically, the PPM fuses features under four different pyramid scales, with bin sizes of 1×1, 2×2, 3×3 and 6×6, respectively. Therefore, the input of the PPM module is the output feature maps of Conv4 layer with size [1024,56,56], where 1024 is the number of channels and the spatial size of the feature maps is 56×56. The output of the PPM module is feature maps of size [128,56,56]. For more implementation details of the PPM module, one can also refer to [26].

### 3.2. Cross-Connection Layer (CCL)

**Figure 3:**
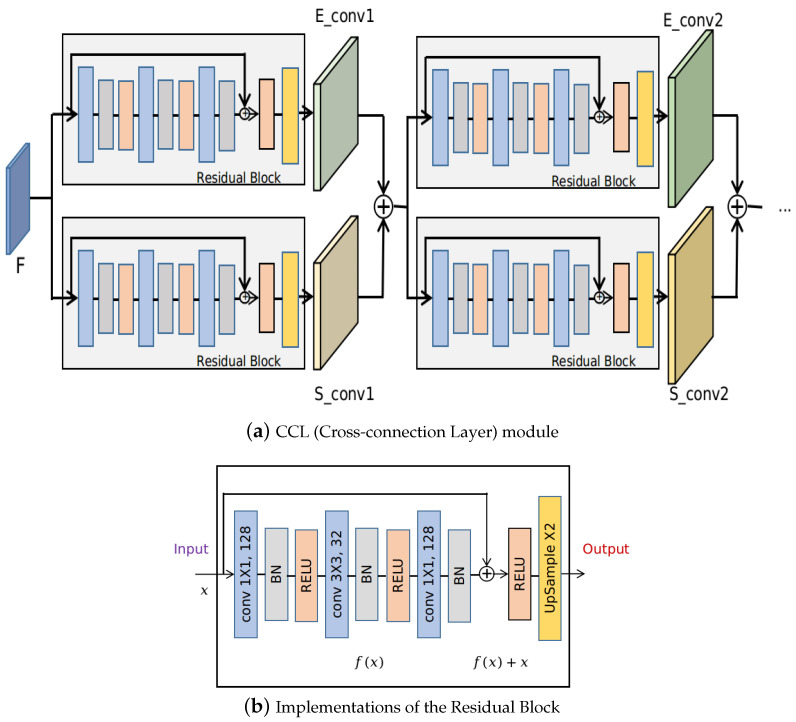
Implementations of the CCL module. The inputs are feature maps F obtained from the backbone CNN. (**a**) shows the implementation details of a Residual block in (**b**), and blocks with the same color indicate the same operation.

The proposed method consists of two parallel branches to predict the edge and segmentation mask. Interactions between these two branches are realized by feeding each task’s intermediate feature maps as the inputs of the next sub-block of the other task, which is referred to as the CCL in this paper. In this case, the edge information is leveraged during the forward prediction of the foreground mask prediction, which can implicitly regularize the mask boundary and make the model focus more on the edges. Similarly, mask information which contains dense pixel information and contextual information are also used to guide the learning of the edges of segmentation masks. Specifically, the layers S_conv1,S_conv2,S_conv3,E_conv1,E_conv2,E_conv3 in CCL are implemented using the residual block as shown in Figure 3a. S_conv1,S_conv2,S_conv3 are the sequential convolutional blocks of the Seg subnet, while E_conv1,E_conv2,E_conv3 are the sequential convolutional blocks of the Edge subnet. Implementation details of a residual block are shown in Figure 3b. The residual block first uses 1×1 kernels to scale the input feature maps. Afterward, 3×3 kernels are used and the number of channels is shrunk to 32. The final output feature maps are of the same size as the input, which is realized by using 1×1 kernels and setting the number of channels to be 128. Upsampling by a factor of 2 is performed after each residual block to increase the resolution of feature maps. The size of the output feature maps at layer S_conv3 and E_conv3 is 448×448, which equals the input image size.

### 3.3. Multi-Scale Feature Aggregation (MSFA)

**Figure 4:**
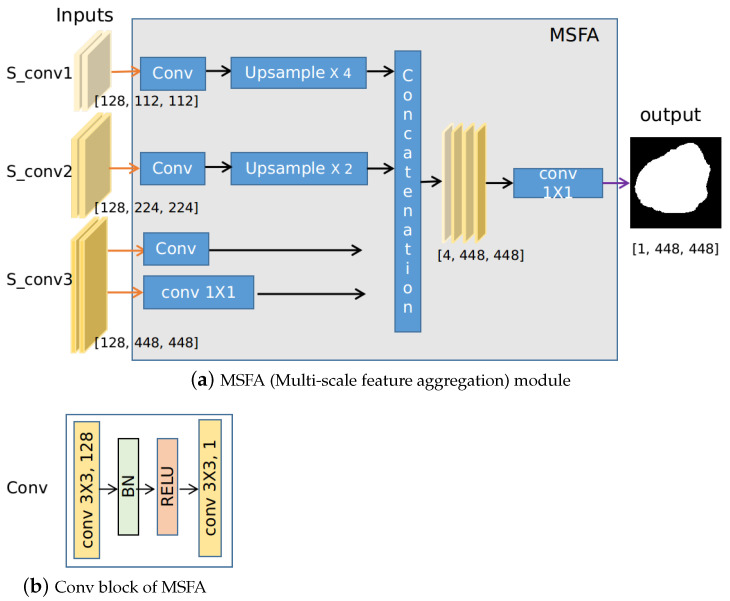
Implementations of the MSFA module, where the segmentation branch is used as an example. The inputs are feature maps of different scales from the segmentation branch, e.g., $S_{conv1},S_{conv2},S_{conv3}$. Four prediction masks are obtained after some convolution and pooling operation. The final output is the weighted sum of these predictions which can be automatically learned by 1×1 convolution.

The MSFA module, shown in Figure 4a, is used to aggregate the feature maps from each resolution and make the final prediction. It first uses the Conv block to generate an output prediction map for feature maps at each resolution, which can be regarded as the process to make predictions at a certain scale. The Conv block consists of standard convolution operations: first, convolution with 3×3 kernels (the number of channels is 128) is performed. Batch normalization (BN) and rectified linear unit (RELU) are used afterward. The output of the Conv is a feature map with depth one, which is obtained by convolving with a 3×3 kernel. For instance, feature maps at layer S_conv1,S_conv2,S_conv3 are fed into the MSFA module and three feature maps are generated at different scales via the Conv block. To leverage the feature maps at a higher resolution more effectively, we also generate the 4th feature map by directly convoluting $S_{conv3}$ with a 1×1 kernel. These four predictions are then upsampled to 448 and are concatenated, resulting in feature maps of size 4×448×448. Finally, these four prediction masks are convolved with 1×1 kernel to aggregate these feature maps into one final prediction. The weight for the prediction of each scale can be automatically learned in this case. Figure 4 shows the schematic of the MSFA module for the Seg subnet, the schematic of the MSFA module for the edge subnet is similar. For both segmentation and edge prediction, shared parameters are used in the MSFA module, which encourages the prediction masks and edges to share a similar quality.

The class-balanced cross entropy loss is used as the cost function for the segmentation and the edge prediction task. The class-balanced cross entropy loss *l* of a prediction is calculated using the following equation:

(1) $$l=-\frac{1}{N}\sum_{n=1}^{N}\left[w_{1}\times y_{n}\times log\left(h_{\theta }\left(x_{n}\right)\right)+w_{0}\times \left(1-y_{n}\right)\times log\left(1-h_{\theta }\left(x_{n}\right)\right)\right]$$

where

*N*: number of pixels;
$y_{n}$: target label for pixel n;
$x_{n}$: input pixel n;
$h_{\theta }$: model with neural network weights θ;
$w_{1}$: weight for foreground pixels;
$w_{0}$: weight for background pixels;

The class balanced weight $w_{1}$ and $w_{0}$ are calculated by inverse class frequency: $w_{1}=\frac{N_{neg}}{N}$ and $w_{0}=\frac{N_{pos}}{N}$. $N_{neg}$ and $N_{pos}$ are the number of background and foreground pixels of a ground truth mask, respectively. A parameter α is used to balance the loss of these two tasks. The final loss function *L* is given by:

(2) $$L=\alpha L_{seg}+L_{edge}$$

where $L_{seg}$ and $L_{edge}$ are the loss for segmentation and edge prediction task over the entire training data, respectively. Both $L_{seg}$ and $L_{edge}$ use Equation (1) to calculate the loss. For the proposed method, we manually set α=0.05 to make the $L_{seg}$ and $L_{edge}$ in a similar range of values during training. The proposed method can be trained in an end-to-end manner.

## 4. Experimental Results

In this section, we first present the implementation details of the proposed technique. The dataset and evaluation metrics used are then described. Experiments regarding the parameter setting and the ablation study are conducted to show the importance of the parameter and each module. Finally, the performance of the technique is evaluated and compared with the state-of-the-art techniques.

### 4.1. Implementation Details

The proposed model is learned using the training data of ISBI2017, and the performance is evaluated on the testing data. For training the proposed model, a dermoscopic image (input), its corresponding ground-truth segmentation mask and edge (contour) image (outputs) are required. The ground truth of the edge image can be automatically obtained from the ground truth of the segmentation mask by contour detection technique. During the training phase, online data augmentation is used to increase the number of training images. Data augmentation techniques, including random horizontal and verticle flipping, center cropping at random scale [0.75, 1.25], random rotation in the degree range [−20, 20], ground truth cropping with zero-padding of 50 pixels, and an image deformation method named Rigid Moving Least Squares [41] are used to generate more training images. All the images are then rescaled to the size of 448×448. For training the proposed neural network, we set the batch size to be 8 and train it for 30 epochs. Adam optimization algorithm with an initial learning rate of 0.0001 is used, which decreases exponentially with a learning rate decay γ=0.9.

During the testing phase, only the segmentation mask is needed to evaluate the performance of the proposed method. Test augmentation is also performed by rotating the input test image by $90^{\circ }$, $180^{\circ }$, $270^{\circ }$, flipping horizontally and vertically. Prediction of the original image can be obtained by reverse operation of the predictions to the transformed images. The average of these prediction outputs is then used as the final segmentation output.

The skin lesion segmentation method is implemented using PyTorch. All the experiments were conducted on a server with an Intel Xeon Processor CPU and two GPUs of Nvidia Tesla V100 with 16 GB memory.

### 4.2. Database

ISBI2017 for skin lesion detection [12] is used for the evaluation of the proposed method. The images are collected from different institutes with different imaging instruments, and are of various sizes, ranging from 767×1022 to 4499×6748. Severe illumination variation and various artifacts (hairs, glue, color-marks, ruler) are witnessed in this dataset. The number of images for the training set, validation set and test set is 2000, 600 and 150, respectively.

### 4.3. Evaluation Metrics

To evaluate the segmentation results, the Jaccard Index (JA), Dice Coefficient (DC), Accuracy (ACC), Sensitivity (SE) and Specificity (SP) are used in this paper. These criteria are defined as follows:

(3) $$\mathrm{JA}=\frac{\mathrm{TP}}{\mathrm{TP}\;+\;\mathrm{FP}\;+\;\mathrm{FN}}$$

(4) $$\mathrm{DC}=\frac{2\cdot \mathrm{TP}}{2\cdot \mathrm{TP}+\mathrm{FP}\;+\;\mathrm{FN}}$$

(5) $$\mathrm{ACC}=\frac{\mathrm{TP}\;+\;\mathrm{TN}}{\mathrm{TP}\;+\;\mathrm{TN}\;+\;\mathrm{FP}\;+\;\mathrm{FN}}$$

(6) $$\mathrm{SEN}=\frac{\mathrm{TP}}{\mathrm{TP}\;+\;\mathrm{FN}}$$

(7) $$\mathrm{SPE}=\frac{\mathrm{TN}}{\mathrm{TN}\;+\;\mathrm{FP}}$$

where TP (True Positive) is the number of foreground pixels being correctly classified as foreground (interest region). TN (True Negative) is the number of background pixels being correctly classified as background (skin region). FP (False Positive) is the number of background pixels being wrongly classified as foreground. FN (False Negative) is the number of foreground pixels being wrongly classified as background. JA represents the ratio of overlapping area and the union area between the predicted segmentation mask and the ground truth mask. DC is twice the overlapping area divided by the total number of pixels in both images. Both metrics reflect how close the prediction mask is to the ground truth mask. ACC represents the percentage of correctly classified pixels among the total number of pixels. SEN represents the proportion of foreground pixels being correctly segmented against the total number of foreground pixels while SPE represents the proportion of background pixels being correctly segmented against the total number of background pixels.

### 4.4. Parameter Setting of the Loss Function

**Figure 5:**
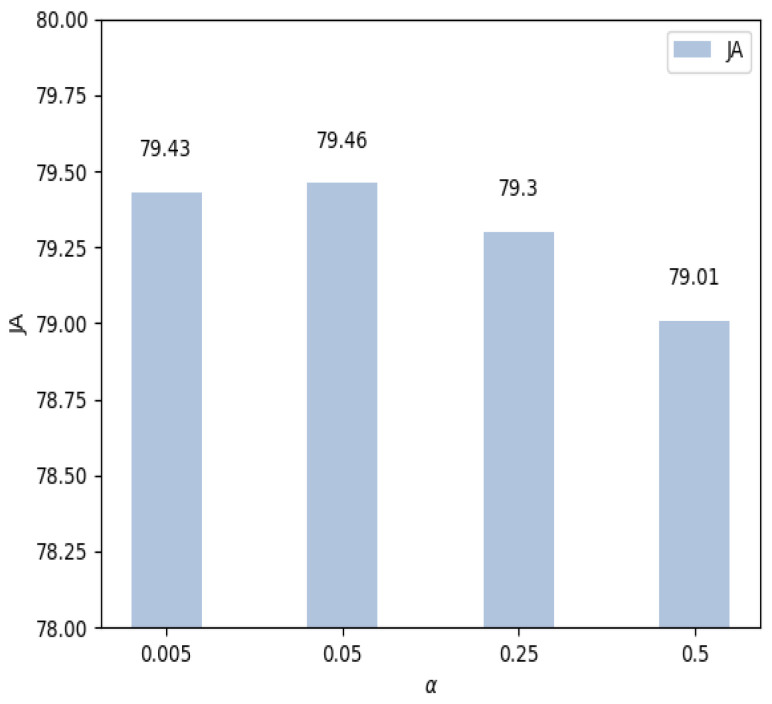
Experiment results with different values of α on the test set.

As in Eqution (2), there is one important parameter α that balances the loss between the segmentation task and the edge prediction task. To investigate the impact of α, experiments regarding different values of the parameter α are conducted. We train the proposed model using the training data of ISBI2017 and evaluate it on the testing data. The parameter α can be critical to obtaining a good segmentation performance. Therefore, experiments using different α with the values of $5\times 10^{-3},0.05,0.25,0.5$ are conducted. Experimental results with different values of α are shown in Figure 5, where JA is used as the evaluation metric. More results are provided in Table 2.

As we can see from Figure 5, α=0.05 presents the best performance regarding the JA. Performance of the proposed method is relatively stable when the values of α are smaller than 0.05, and it gradually decreases with increasing values of α. Especially, the JA drops from 79.46 to 79.01 when the value of α increases from 0.05 to 0.5. This is consistent with the motivation of the proposed method since a larger value of α indicates a weaker role of edge prediction. In the extreme case, only the segmentation branch is updated and learned during training when the parameter α is large enough. Therefore, in this paper, we have used α=0.05 to obtain a balanced segmentation loss and edge prediction loss. Experimental results in Table 2 have also verified that the edge prediction can help the segmentation task. The segmentation branch will focus more on the boundaries of the pigment regions, which is usually crucial for successful segmentation.

### 4.5. Ablation Study

To show the effectiveness of the proposed method, an ablation study about the proposed method on the ISBI2017 test data is conducted. We name the three yellow blocks (in Figure 2) in the segmentation branch as the Seg Subnet. Similarly, the three green blocks in the edge prediction branch are named the Edge Subnet. Results of the ablation study are shown in Table 3. ResNet + PPM + Seg is the backbone CNN with a Seg subnet for the segmentation task, which can be regarded as a strong baseline model for the skin lesion segmentation task. JA is regarded as the main evaluation metric for the segmentation task as in the literary works, which reflects the percentage of overlap between the prediction mask and the ground-truth mask. A JA value of 77.01% is obtained for this baseline method. By adding the Edge subnet, we further increase the JA by 0.57%, which verifies that the auxiliary task (edge prediction) can benefit the segmentation mask. Our final model is the proposed method with the Seg subnet, the Edge subnet and the MSFA module, which obtains the best performance with a JA value of 79.46. An increase of 2.45% JA value is observed for the proposed method compared with the baseline method, which verifies the effectiveness of the proposed method. In addition to JA, the proposed method provides an improvement of 1.16%, 1.92%, and 1.39% for ACC, DC and SP over the baseline method.

### 4.6. Comparison with State-of-The-Art Methods

In this section, we compare the proposed method with other state-of-the-art methods using the ISBI2017 test data. The experimental results are shown in Table 4. Since ISBI2017 is a challenge dataset for skin lesion segmentation, ensembling techniques by using different CNN models [34], post-processing [32,34,36] are widely used. In comparison, our method only learns one model in an end-to-end manner without any pre-processing and post-processing methods and still achieves the best performance with a JA of 79.46, ACC of 94.32, SEN of 88.76.

## 5. Discussion

**Figure 6:**
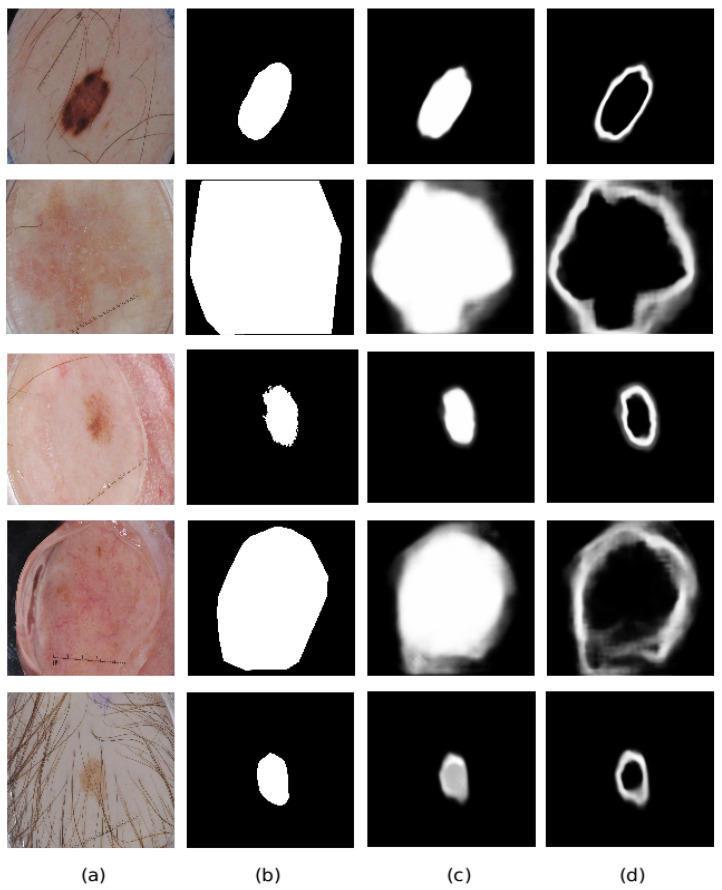
Output visualization of the proposed method. (**a**) input test image; (**b**) the corresponding ground truth segmentation mask; (**c**) the output probability map of the segmentation prediction branch; (**d**) the output probability map of the edge prediction branch.

In this section, a qualitative analysis of the performance of the proposed method is also conducted. The final outputs of the segmentation and edge prediction branches are displayed in Figure 6. As shown in Figure 6, the proposed method can segment the pigment regions correctly in most cases. The first row displays the output predictions given an input image, which is an easy case since the color contrast of the input image is high between the foreground and the background region. Therefore, the proposed method can detect the pigment region with high accuracy. The second, third and fourth rows present the images with fuzzy boundaries and low contrast. In addition, the existence of glue is also observed among these images, which will make it extremely difficult to identify the boundaries. Output probability maps of the segmentation and edge prediction branches become slightly fuzzy on the boundaries in these cases, but still with decent results. The bottom row shows an input image with hairs and low contrast, which can affect the textures of the skin lesions and prohibit successful learning. Nevertheless, the proposed method still successfully segments the pigment regions. In other words, the proposed method is robust to noisy items and obtains an overall good performance.

**Figure 7:**
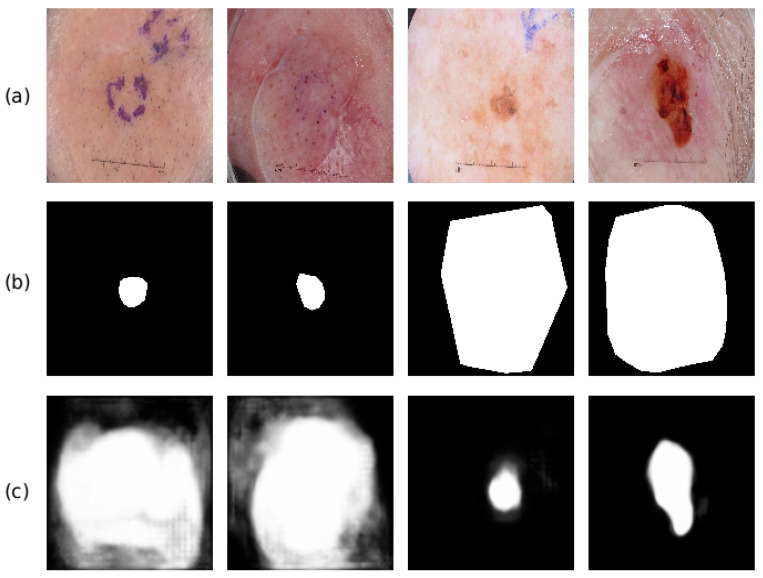
Some examples about the failed cases for the proposed method. (**a**) the input test images; (**b**) the corresponding ground truth segmentation masks; (**c**) the probability map of the proposed method.

Figure 6 has shown some example images that have been successfully segmented. To further analyze the performance of the proposed method, we also display some failed cases in Figure 7. The first row displays the input test images, the second row shows the corresponding ground truth segmentation mask, and the third row is the prediction probability map of the segmentation mask. As we can see from the first two columns of Figure 7, the proposed method generates larger feature maps than the ground truth masks, which is mainly due to the existence of the glue and the unclear boundaries. They will distract the proposed method and deteriorate the segmentation performance. In contrast, outputs in the third and fourth columns show that the proposed method predicts smaller segmentation masks than the ground truth masks. The learned model tends to treat the dark area as the foreground regions (which is the most frequent cases for dermoscopic images), and it fails when there are multiple colors scattered within the same lesions in some difficult cases. For instance, the proposed method fails when the foreground region contains a dark area surrounded by an area with light color, whose appearance is more similar to the healthy skin region (see the last two columns of Figure 7). Such phenomenon has also been found in previous works [42,43], which may be caused by the scarce samples and it will be the focus of our future research. It is also worth noting that the ground truths of the segmentation masks of ISBI2017 are labeled using different methods (e.g., manual labeling, thresholding methods, interactive labeling methods). Therefore, there are annotation disagreements among the labeled images, which have been described in [44,45] and may hinder the learning of the proposed method.

## 6. Conclusions

In this paper, a novel convolutional neural networks (CNN) based method with auxiliary task learning is proposed. Edge prediction, as an auxiliary task, is performed simultaneously with the segmentation prediction to help the segmentation task. The ground truth of the edge prediction task can be obtained automatically from the ground truth segmentation masks by using a standard contour detection method, and hence no extra labeling effort is required. A cross-connected layer (CCL) module is proposed, where the intermediate feature maps of each task are fed into the other task’s subblock, which implicitly guides the neural networks to focus on the boundary region and boosts the performance of the segmentation task. A multi-scale feature aggregation (MSFA) module is proposed, which can automatically learn the final mask by aggregating the output of different scales. An ablation study has shown the benefits of these proposed modules. Experimental results with the ISBI2017 dataset have shown that the proposed method outperforms the other state-of-art methods in terms of performance measures such as the Jaccard index and accuracy.

## Figures and Tables

**Table 1:** Architecture of the proposed method. The input image size is 448×448.

Layer Name	Output Size	Output Channel Dimension	Operations
Conv1	224×224	64	$\left[\begin{array}{c} 7\times 7,\;64,\;\mathrm{stride}\;2\\ 3\times 3,\;\max\;\;\;\mathrm{pool},\;\;\;\mathrm{stride}2 \end{array}\right]$
Conv2	112×112	256	$\left[\begin{array}{c} 1\times 1,\;64\\ 3\times 3,\;64\\ 1\times 1,\;256 \end{array}\right]\times 3$
Conv3	56×56	512	$\left[\begin{array}{c} 1\times 1,\;128\\ 3\times 3,\;128\\ 1\times 1,\;512 \end{array}\right]\times 4$
Conv4	56×56	1024	$\left[\begin{array}{c} 1\times 1,\;256\\ 3\times 3,\;256\\ 1\times 1,\;1024 \end{array}\right]\times 23$
*F*	56×56	128	PPM [26]
E_conv1/S_conv1	112×112	128	$\left[\begin{array}{c} 1\times 1,\;128\\ 3\times 3,\;32\\ 1\times 1,\;128 \end{array}\right]\times 1$
E_conv2/S_conv2	224×224	128	$\left[\begin{array}{c} 1\times 1,\;128\\ 3\times 3,\;32\\ 1\times 1,\;128 \end{array}\right]\times 1$
E_conv3/S_conv3	448×448	128	$\left[\begin{array}{c} 1\times 1,\;128\\ 3\times 3,\;32\\ 1\times 1,\;128 \end{array}\right]\times 1$

**Table 2:** Experimental results with different values of α on the test set.

α	ACC	DC	SEN	SP	JA
0.005	94.17	**87.14**	88.77	95.56	79.43
0.05	94.32	87.13	88.76	**96.51**	**79.46**
0.25	**94.33**	87.09	88.06	96.40	79.30
0.5	94.11	86.78	**89.25**	93.39	79.01

The best performance corresponding to each metric is shown in bold.

**Table 3:** Ablation study of the proposed method.

Method	ACC	DC	SEN	SP	JA
ResNet + PPM + Seg	93.16	85.21	**88.87**	95.12	77.01
ResNet + PPM + Seg + Edge	93.54	85.66	87.11	96.61	77.58
Proposed	**94.32**	**87.13**	88.76	**96.51**	**79.46**

The best performance corresponding to each metric is shown in bold.

**Table 4:** Experimental results compared with state-of-the-art methods on ISBI2017 test data.

Method	ACC	DC	SEN	SP	JA
Liu et al. [32]	93.00	84.00	82.90	98.00	75.20
Abhishek et al. [33]	92.22	83.86	87.06	95.16	75.70
Yuan et al. [34]	93.40	84.90	82.50	97.50	76.50
AI-Masni et al. [35]	94.03	87.08	85.40	96.69	77.11
Bi et al. [36]	94.08	85.66	86.20	96.71	77.73
Sarker et al. [37]	93.60	**87.80**	81.60	**98.30**	78.20
Proposed	**94.32**	87.13	**88.76**	96.51	**79.46**

The best performance corresponding to each metric is shown in bold.

## Data Availability

Not applicable.
